# An Exploration on Greenhouse Gas and Ammonia Production by Insect Species Suitable for Animal or Human Consumption

**DOI:** 10.1371/journal.pone.0014445

**Published:** 2010-12-29

**Authors:** Dennis G. A. B. Oonincx, Joost van Itterbeeck, Marcel J. W. Heetkamp, Henry van den Brand, Joop J. A. van Loon, Arnold van Huis

**Affiliations:** 1 Laboratory of Entomology, Department of Plant Sciences, Wageningen University, Wageningen, The Netherlands; 2 Adaptation Physiology Group, Wageningen Institute of Animal Sciences, Wageningen University, Wageningen, The Netherlands; New Mexico State University, United States of America

## Abstract

**Background:**

Greenhouse gas (GHG) production, as a cause of climate change, is considered as one of the biggest problems society is currently facing. The livestock sector is one of the large contributors of anthropogenic GHG emissions. Also, large amounts of ammonia (NH_3_), leading to soil nitrification and acidification, are produced by livestock. Therefore other sources of animal protein, like edible insects, are currently being considered.

**Methodology/Principal Findings:**

An experiment was conducted to quantify production of carbon dioxide (CO_2_) and average daily gain (ADG) as a measure of feed conversion efficiency, and to quantify the production of the greenhouse gases methane (CH_4_) and nitrous oxide (N_2_O) as well as NH_3_ by five insect species of which the first three are considered edible: *Tenebrio molitor*, *Acheta domesticus*, *Locusta migratoria*, *Pachnoda marginata,* and *Blaptica dubia.* Large differences were found among the species regarding their production of CO_2_ and GHGs. The insects in this study had a higher relative growth rate and emitted comparable or lower amounts of GHG than described in literature for pigs and much lower amounts of GHG than cattle. The same was true for CO_2_ production per kg of metabolic weight and per kg of mass gain. Furthermore, also the production of NH_3_ by insects was lower than for conventional livestock.

**Conclusions/Significance:**

This study therefore indicates that insects could serve as a more environmentally friendly alternative for the production of animal protein with respect to GHG and NH_3_ emissions. The results of this study can be used as basic information to compare the production of insects with conventional livestock by means of a life cycle analysis.

## Introduction

Production of greenhouse gasses (GHG) is considered as an important cause of climate change [1], [2]. The most important GHGs are carbon dioxide (CO_2_), methane (CH_4_) and nitrous oxide (N_2_O). Since the end of the 18^th^ century the atmospheric carbon-dioxide concentration has increased by 30% and CH_4_ concentrations by 50% [3]. CH_4_ and N_2_O have considerably greater global warming potentials (GWPs) than CO_2_. By assigning CO_2_ a value of 1 GWP, the warming potentials of these other gases can be expressed on a CO_2_-equivalent basis: CH_4_ has a GWP of 25, and N_2_O has a GWP of 298 [1]. The relative contribution of CO_2_ equivalents (CO_2_ eq.) of the livestock sector is large, amounting up to 18% of total anthropogenic GHG emissions [2]. Based on a Life Cycle Analysis (LCA) that takes the entire production process of animal products into account, the global contribution to GHG emissions by the animal sector are: 9% for CO_2_ (fertilizer production for feed crops, on-farm energy expenditures, feed transport, animal product processing, animal transport, and land use changes), 35–40% for CH_4_ (enteric fermentation in ruminants and from farm animal manure) and 65% for N_2_O (farm manure and urine) [2]. Direct CO_2_ production through respiration is not relevant when determining the impact of GHGs as respiration by livestock is not considered a net source of CO_2_ [2]. The respired carbon, which comes from the feed, was first taken up from CO_2_ in the air and stored in an organic compound during the production of the feed. However, the ratio between body growth realised and CO_2_ production is an indicator of feed conversion efficiency and thereby a relevant indicator for the environmental impact [4].

Livestock is also associated with environmental pollution due to ammonia (NH_3_) emissions from manure and urine, leading to nitrification and acidification of soil [5]. Although not considered a GHG, NH_3_ can indirectly contribute to N_2_O emission [2], as conversion takes place by specialized soil bacteria [6]. Livestock is estimated to be responsible for 64% of all anthropogenic NH_3_ emissions [2]. The main source of gaseous NH_3_ is bacterial fermentation of uric acid in poultry manure [7], [8] and bacterial fermentation of urea in mammals [9]. Besides these environmental problems the livestock sector faces challenges regarding resistance to antibiotics, zoonosis and animal welfare [10].

All these problems together illustrate the need to find alternatives for conventional sources of animal protein. Mini-livestock, for instance edible insects, have been suggested as an alternative source of animal protein [11]. Production of animal protein in the form of edible insects supposedly has a lower environmental impact than conventional livestock [12], [13], [14]. When evaluating the total environmental impact of animal protein production, a LCA, in which all production factors are taken into account, is needed. Differences in environmental impact in a LCA can be explained mainly by three factors: enteric CH_4_ emissions, feed conversion efficiencies and reproduction rates [4].

Before performing a LCA, it is necessary to know the GHG production by edible insects. This information is lacking in literature. Therefore, in this study we experimentally quantified the direct production of the GHGs CH_4_ and N_2_O for five insect species. CO_2_ production and average daily gain (ADG) were quantified to provide an estimation of feed conversion efficiency. Additionally, NH_3_ emissions were quantified. The results of this study represent a quantification of the insect physiological contribution to GHG production by insects and can in turn be used to create a LCA for insect-derived products.

## Materials and Methods

### 2.1 Animals and housing

Five insect species were studied: fifth larval stage mealworms *Tenebrio molitor* L. (Coleoptera: Tenebrionidae), fifth and sixth nymphal stage house crickets *Acheta domesticus* (L.) (Orthoptera: Gryllidae), third and fourth stage nymphs of migratory locusts *Locusta migratoria* (L.) (Orthoptera: Acrididae), third larval stage sun beetles *Pachnoda marginata* Drury (Coleoptera; Scarabaeidae) and a mix of all stages of the Argentinean cockroach *Blaptica dubia* (Serville) (Dictyoptera: Blaberidae). Currently, *T. molitor*, *A. domesticus* and *L. migratoria* are considered edible, while *P. marginata* and *B. dubia* are not. The latter two species were included since they are a potential source of animal protein, for instance by means of protein extraction. These two species can be bred in large numbers with little time investment and are able to utilise a wide range of substrates as feed [15], [16].

Per species three to six repetitions were conducted each for a period of three days. Animals were housed per species in two cages or containers per respiration chamber. These containers were placed in one of two, identical, open circuit climate respiration chambers measuring 80*50*45 cm, with a total volume of 265 L [17]. Within these climate respiration chambers, *T. molitor* and *P. marginata* were housed in two stacked plastic containers (50*30*8.7 cm). The three other species were housed in metal wire cages (45*37.5*41 cm; mesh width 1 mm) with a glass cover plate. To increase surface area for *A. domesticus* and *B. dubia*, hollow plastic tubes (20 cm long and 3 cm in diameter), were stacked to a height of 30 cm in the wired cages, while for *L. migratoria*, two V-shaped-folded metal screens (70*15 cm) were entered per cage. Humidity, temperature, and day length were based on rearing conditions used by commercial insect rearing companies (Table 1). All animal masses reported are averages of fresh mass per cage. The starting and final animal mass per cage are provided in Table 1.

**Table 1:** Mean values and standard deviations of temperature, humidity, ventilation, hours of light per day and average start and final weight for five insect species.

	Pachnoda. marginata	Tenebrio molitor	Blaptica dubia	Acheta domesticus	Locusta migratoria
Temperature (°C)	28.0±0	25.0±0	28.0±0	28.0±0	32.0±0
Humidity (%)	84.3±3.3	79.8±0.2	70.0±0.0	69.9±0.1	69.7±0.2
Ventilation (L/min)	6.46±2.06	6.82±1.31	5.16±0.05	11.18±1.80	4.98±0.39
Hours of light per day	0	0	12	12	12
Start weight (kg)	0.99	0.91	1.10	0.96	0.08
Final weight (kg)	1.10	1.10	1.28	1.17	0.13

### 2.2 Diet

Food was provided for each species at the beginning of each repetition, except when mentioned otherwise.

*Tenebrio molitor* larvae were reared in 300 g mixed grain substrate (wheat, wheat bran, oats, soy, rye and corn, supplemented with beer yeast) with on top pieces of carrot (±15*2 cm) weighing a total average of 637 g per repetition.

*Acheta domesticus* was provided with chicken mash (501 g) with carrot pieces (784 g) on top for each repetition.

*Locusta migratoria* was provided with wheat bran (70 g; Arie Blok Animal Nutrition, Woerden, The Netherlands) in a metal bowl at the beginning of each repetition. Fresh Perennial ryegrass (*Lolium perenne)* was provided daily (463 g in three days). The grass was grown by Unifarm, Wageningen University and Research centre, Wageningen, The Netherlands.

*P. marginata* larvae were kept in a peat moss substrate (2.0 kg per respiration chamber) in which chicken mash (285 g) was mixed at the beginning of each three-day repetition. Pieces of carrot (±15*2 cm) with an average total mass of 161 g per repetition were put on top of the substrate.

*B. dubia* was provided with a chicken mash diet (199 g) and carrots (559 g), fresh carrot being added during the repetitions.

Peat moss, chicken mash, and carrots, offered to *A. domesticus*, *P. marginata* and *B. dubia* were provided by Kreca V.O.F, Ermelo, The Netherlands. The carrots and mixed grains substrate offered to *T. molitor* were provided by Insectra, Deurne, The Netherlands.

### 2.3 Gas measurements

During the experiment concentrations of CO_2_ and CH_4_ were measured every 9 min in the ingoing and outgoing air stream of the respiration chambers. The difference in CO_2_ and CH_4_ concentrations between ingoing and outgoing air thus represents the total production of CO_2_ and CH_4_ of insects, feed, and substrate. The exact air volumes were measured with a calibrated Schlumberger G1.6 dry gas meter and corrected for measured air temperature and pressure. CO_2_ and CH_4_ concentrations were measured in dried gas. Gas was dried in a +2°C dew-point cooler. Nondispersive infrared analyzers were used to measure CO_2_ (type Uras 3G, Hartmann and Braun, Frankfurt, Germany) and CH_4_ (type Uras 10E, Hartmann and Braun, Frankfurt, Germany). The refreshed air volume was set so that CO_2_ levels did not exceed 1%. From each climate respiration chamber, as well as from the incoming air, an air sample was taken for N_2_O analysis after 24, 48, and 72 h with a 60 ml syringe. The syringes were sealed by a shutoff valve and stored at 20°C until analysis (within 48 h). The N_2_O concentration was analysed by a gas chromatograph (CE instruments GC8000 Top, Interscience, Breda, The Netherlands) using a Haysep Q 80–100 mesh 2 m×1/8″ SS column, at a constant temperature of 60°C. N_2_O was detected with an electron capture detector (ECD). Injection volume was 5.0 ml in a fixed loop.

NH_3_ concentrations in the climate respiration chambers were determined twice daily (at 12.00 and 24.00 h) by means of a gas detection tube system (Kitagawa, type AP-20; Komyo rikagaku kogyo, Tokyo, Japan; type 105 NH_3_ gas detector tubes with a range of 1–20 ppm).

### 2.4 Calculations

Production of N_2_O was calculated by subtracting the N_2_O concentration from the incoming air from that in the outgoing air. These differences were then used in a formula adapted from Wheeler et al (2003) [18]:

ER  =  Emission rate of N_2_O  =  [N_2_O] change (ppm×10^−6^)×VV (m^3^/day)×44 (g/mol)/0.0224 (m^3^/mol), where VV =  ventilation volume of air in a specified time period. The average concentration difference of the three samples taken during the three-day period was used to determine the average N_2_O production in a repetition.

The formula used by Wheeler (2003) was also used for the calculation of NH_3_ production. A molecular mass of 17 was used and instead of a difference in concentration, the measured concentration was used, leading to a slight overestimation of the actual NH_3_ production (between 0 and 0.1 mg/kg BM/day).

CO_2_ equivalents were calculated by adding the multiplications of the produced amounts of CH_4_ and N_2_O with their global warming potential; 25 for CH_4_, and 298 for N_2_O [1].

Mean body mass was calculated by averaging the body mass at the start of the experiment and the body mass at the end of the experiment. Average daily gain (ADG) was calculated as follows: (((End mass - Start mass)/Start mass)/3)*100%, in which 3 is the number of days the experiment was running.

The ratio between CO_2_ production per unit biomass per day and ADG gives an indication of the feed conversion efficiency, in which higher values indicate lower efficiencies.

To determine CO_2_ production from feed and substrate, all feeds were independently tested in the same respiration chambers, without the animals. A linear time course of consumption was assumed and CO_2_ production was recalculated to kg of live insect.

### 2.5 Statistics

The N_2_O and NH_3_ assay data were subjected to a two-way analysis of variance (ANOVA) with species and time of sampling (24, 48, or 72 h) as fixed factors to determine whether the time of sampling had an effect. No significant effect of the time of sampling was found for N_2_O (Pillai's trace: F = 1.467, P = 0.199). Therefore, the average of the three samples taken during the 3-day trial period was used to determine the change per repetition and to calculate total production. However, NH_3_ production was significantly affected by the time of sampling (day or night; Pillai's trace: F = 4.065, P = 0.019) and the day of the repetition (first, second or third; Pillai's trace: F = 17.170, P<0.001). CO_2_ and CH_4_ production for all five species were analyzed by means of a one way analysis of variance (ANOVA) followed by a Tukey post hoc test. Statistical analysis of all data was done by means of SPSS 15.0.

## Results

Production of CO_2_ is expressed per kilogram of mean live body mass (BM) per day (24 hours) and per kilogram of mass gain (Table 2) and the average daily gain (ADG) is reported (Table 2). Production of CH_4_, N_2_O, CO_2_ equivalents, and NH_3_, are expressed per kilogram of mean live body mass (BM) per day (Table 3) and per kilogram of mass gain (Table 4).

**Table 2:** CO_2_ production (average ± standard deviation) per kilogram of bodymass per day, per kg of mass gain and average daily gain for five insect species, pigs and beef cattle.

Species	CO_2_ (g/kg BM/day)	CO_2_ (g/kg mass gain)	ADG (%)
*Pachnoda marginata (n = 4)*	50±22 ^a^	1,539±518^ a^	4.0±2.1%^ a^
*Tenebrio molitor (n = 4)*	61±9^ b^	1,031±349^ b^	7.3±2.5%^ b^
*Blaptica dubia (n = 3)*	19±3^ c^	337±51 ^c^	6.1±0.7%^ c^
*Acheta domesticus (n = 4)*	68±10^ d^	1,468±971^ a^	7.2±3.4%^ b^
*Locusta migratoria (n = 6)*	110±21^ e^	734±119 ^d^	19.6±2.1%^ d^
Pigs	21.6–29.6	865–1,194	3.2±0.53%
Beef cattle	5.3–7.0	2,835	0.3±0.07%

BM = Body Mass;

ADG = Average daily gain;

Reported values for pigs and beef cattle were obtained from: [5] Aarnink et al., 1995; [49] Groot Koerkamp et al., 1998; [52] Demmers et al., 2001; [50] Nicks et al., 2003; [59] Beauchemin & McGinn, 2005; [48] Cabaraux et al., 2009 and [53] Harper et al., 2009. Mean values bearing different superscripts in a column differ significantly (P<0.05).

**Table 3:** CH_4_, N_2_O, CO_2_ eq. and NH_3_ production (average ± standard deviation) per kilogram of bodymass per day for *five insect species*, pigs and beef cattle.

Species	CH_4_ (g/kg BM/day)	N_2_O (mg/kg BM/day)	CO_2_ eq. (g/kg BM/day)	NH_3_ (mg/kg BM/day)
*Pachnoda marginata (n = 4)*	0.16±0.085 ^a^	0.0±0.03^ a^	4.00±2.13^ a^	0.1±0.16 ^a^
*Tenebrio molitor (n = 4)*	0.00±0.002 ^b^	1.5±0.13^ b^	0.45±0.04^ b^	0.0±0.09^ a^
*Blaptica dubia (n = 3)*	0.08±0.021^ c^	0.3±0.24^ a^	2.12±0.57^ c^	3.0±1.63^ b^
*Acheta domesticus (n = 4)*	0.00±0.002^ c^	0.1±0.13^ a^	0.05±0.04^ b^	5.4±3.40^ c^
*Locusta migratoria (n = 6)*	0.00±0.017^ c^	8.0±13.50^ b^	2.37±4.02 ^c^	5.4±1.65^ c^
Pigs	0.049–0.098	2.7–85.6	2.03–27.96	4.8–75
Beef cattle	0.239–0.283	N/A	5.98–7.08	14–170

BM = Body Mass;

N/A = Not Available;

Reported values for pigs and beef cattle were obtained from: [5] Aarnink et al., 1995; [49] Groot Koerkamp et al., 1998; [52] Demmers et al., 2001; [50] Nicks et al., 2003; [59] Beauchemin & McGinn, 2005; [48] Cabaraux et al., 2009 and [53] Harper et al., 2009. Mean values bearing different superscripts in a column differ significantly (P<0.05).

**Table 4:** CH_4_, N_2_O, CO2 eq. and NH_3_ production (average ± standard deviation) per kilogram of mass gain for five insect species, pigs and beef cattle.

Species	CH_4_ (g/kg mass gain)	N_2_O(mg/kg mass gain)	CO_2_ eq. (g/kg mass gain)	NH_3_ (mg/day/kg mass gain)
*Pachnoda marginata (n = 4)*	4.9±1.96^ a^	1.03±1.06^a^	121.86±49.09 ^a^	3±4.8^ a^
*Tenebrio molitor (n = 4)*	0.1±0.03^ b^	25.5±7.70^ b^	7.58±2.29^ b^	1±2.0^ a^
*Blaptica dubia (n = 3)*	1.4±0.30^ c^	5.7±4.05^ a^	37.54±8.01^ c^	54±31.1 ^a^
*Acheta domesticus (n = 4)*	0.0±0.09^ b^	5.3±6.05^ a^	1.57±1.80 ^d^	142±184.5^ b^
*Locusta migratoria (n = 6)*	0.0±0.11^ b^	59.5±104.8^ c^	17.72±31.22 ^e^	36±10.8^ a^
Pigs	1.92–3.98	106–3457	79.59–1,130	1140–1920
Beef cattle	114	N/A	2,850	N/A

BM = Body Mass;

N/A = Not Available;

Reported values for pigs and beef cattle were obtained from: [5] Aarnink et al., 1995; [49] Groot Koerkamp et al., 1998; [52] Demmers et al., 2001; [50] Nicks et al., 2003; [59] Beauchemin & McGinn, 2005; [48] Cabaraux et al., 2009 and [53] Harper et al., 2009. Mean values bearing different superscripts in a column differ significantly (P<0.05).

### 3.1 ADG and CO_2_ production

ADG varied between 4.0% (*P. marginata*) and 19.6% (*L. migratoria*) with the three other species having an ADG of 6–7%. CO_2_ production among the five insect species differed significantly and ranged from 19 (*B. dubia*) to 110 (*L. migratoria*) g per kg BM/day. Also, the CO_2_ production per kg of metabolic weight (i.e. the weight of metabolically active body tissue) differed greatly between species (Table 5). CO_2_ production expressed per kg of mass gain was intermediary for *L. migratoria* due to the high ADG. Still, the CO_2_ production of *L. migratoria* per kg of mass gain was more than double the production of CO_2_ by *B. dubia*. *Pachnoda marginata* had the highest production of CO_2_ per kg of mass gain (1,539 g/kg), which was more than double the amount of *L. migratoria*.

**Table 5:** CO_2_ production (g) per kilogram of metabolic weight per day for five insect species, pigs and beef cattle based on Kleiber's law (B = aM^b^).

Species	b = 0.67	b = 0.75	b = 0.82
*Pachnoda marginata (n = 4)*	7	11	17
*Tenebrio molitor (n = 4)*	3	7	12
*Blaptica dubia (n = 3)*	2	4	6
*Acheta domesticus (n = 4)*	4	8	14
*Locusta migratoria (n = 6)*	9	17	29
Pigs	63	50	41
Beef cattle	50	31	21

### 3.2 CH_4_

Production of methane was detected for *P. marginata* and *B. dubia*, but not for the three other species. *Pachnoda marginata* produced more than three times as much CH_4_ per kg of mass gain than *B. dubia* (4.9 *vs* 1.4 g). This difference was caused by a higher production of CH_4_ per kg BM (0.16 g *vs* 0.08 g) and a lower ADG (4.0% *vs* 6.1%).

### 3.3 N_2_O

N_2_O was produced only in significant amounts by *T. molitor* and *L. migratoria* (1.5 and 8.0 mg/kg BM/day, respectively). Production of N_2_O by *L. migratoria* per kg BM was more than 5-fold the production by *T. molitor*, this difference decreased to almost 2.5-fold when expressed per kg of mass gain, due to a much higher ADG of *L. migratoria*.

### 3.4 NH_3_

NH_3_ was produced by *A. domesticus*, *L. migratoria*, and *B. dubia* (3.0–5.4 mg/kg BM/day), and ranged from 36–142 mg/kg of mass gain (Table 3 and 4). Significant differences (Pillai's trace: F = 4.065, P = 0.019) between daytime (12.00) and night-time (24.00) NH_3_ emission levels were found for *A. domesticus* (6.4 and 4.4 mg/kg BM/day), *L. migratoria* (5.6 and 3.9 mg/kg BM/day), and *B. dubia* (3.4 and 2.6 mg/kg BM/day).

## Discussion

Insects, being poikilotherms, do not use their metabolism to maintain a body temperature within narrow ranges, contrary to homeothermic animals. This is expected to result in higher feed conversion efficiencies. CO_2_ production related to growth, has an inverse relationship with feed conversion efficiency in a given situation. CO_2_ production by insects depends on the species, stage of development [19], [20], temperature [21], feeding status [22], and on activity level [23], [24]. A production of 37 g CO_2_/kg BM/day was reported for *Anabrus simplex* (Orthoptera, Tettigoniidae), 40 g CO_2_/kg BM/day for the locust *Schistocerca americana* (Orthoptera; Acrididae) [25] and 94 g/kg BM/day for adult *Tribolium castaneum* (Coleoptera; Tenebrionidae) [26]. All five species in the current study had a fairly high production of CO_2_. This might to a large extent be explained by *ad libitum* feeding during the experiment that has been reported to increase oxygen consumption fivefold [22]. Reported CO_2_ production for inactive, unfed, Tenebrionid adults ranged between 5.4–13.3 g/kg BM/day [27], which is 5–10 times lower than observed for *T. molitor* in this experiment. This can partially be explained by the locomotory activities of *T. molitor* larvae in this experiment [37]. Furthermore, growing larvae are expected to have a higher CO_2_ production than adults. The range of CO_2_ production for *T. molitor* is comparable to the factorial metabolic scope reported for tiger beetles (*Cicindela* spp: Coleoptera; Cicindelidae) of 6.1–16.5 [28].

Size differences in animals account for a difference in metabolic rate, and thereby CO_2_ production. The relation between metabolic rate (B) and body mass (M) was described by Kleiber [29] as B = aM^b^, in which a is a constant and b = 0.75. The value of b has been much debated since [30], [31], [32]. For poikilotherms values between 0.67 and 1.0 have been reported and a comparison of several arthropod species suggested b approximates 0.82 [33], [34]. The value chosen for b has a large impact on the metabolic weight and thereby the calculated CO_2_ production (Table 5). Applying b = 0.75 for pigs and beef cattle and b = 0.82 for insects, resulted in a lower CO_2_ production based on metabolic weight for the studied insect species (Table 5). For *L. migratoria* CO_2_ production was only slightly lower than for beef cattle, however, for the other four species production was between 18% and 54% of that for beef cattle and between 11% and 34% of the CO_2_ production of pigs.

The CO_2_ production per kg BM of insect species investigated in this study was higher than for pigs or cattle (Table 3). This concurs with Prothero *et al*. (1979) [35], who reported a higher oxygen consumption per kg of BM for insects than for mammals, assuming the respiratory quotient (CO_2_ production/O_2_ consumption) has similar values (0.7–1.0) for both animal groups. However, the CO_2_ production per kg of mass gain for the five insect species in the current study (337–1,539 g/kg) was either 39% (minimum values) or 129% (maximum values) when compared with pigs (865–1,194 g/kg) and much lower (12%–54% respectively) than cattle (2,835 g/kg). Therefore, CO_2_ production per kg of mass gain suggests higher feed conversion efficiencies for insects than for mammalian livestock. These results concur with those of other authors [13], [14], [36], [37].

A similar trend was visible for ADG; the ADG for the five insect species studied was 4.0–19.6%, the minimum value of this range being close to the 3.2% reported for pigs, whereas the maximum value was 6 times higher. Compared to cattle (0.3%), insect ADG values were much higher. In general, the rate of ADG depends, amongst others, on life phase. Therefore, where available, literature data on growing animals were used. The fundamental biological differences in growth and development processes between pigs and cattle and the studied insects impeded further synchronization.

CH_4_ production for the species studied was in agreement with Hackstein and Stumm (1994) [38]; for insects, only representatives of cockroaches, termites, and scarab beetles produce CH_4_. This originates from bacterial fermentation by methanobacteriaceae in the hindgut [39].

We found large variability for the N_2_O emission rates. Earlier studies in laying hens using a similar method for determining N_2_O production, concluded that production was either negligible or undetectable [7], [40]. However, other authors [41], [42] determined a production of 28 mg N_2_O/kg BM/day and 52 mg N_2_O/kg BM/day, respectively, indicating the difficulty of accurately determining N_2_O production [43].

In earlier studies respiration of feed was considered to have a negligible effect on utilisation of dry mass as determined gravimetrically [44] and therefore on CO_2_ production. Later studies suggested that respiration by plant leaves can be an important source of error in the calculation of insect feed intake using gravimetric methods [45] and can cause major errors in energy budget studies of plant-feeding insects [46]. Our reported CO_2_ production includes the respiration of the feed (Table 6). The extremely high contribution to total CO_2_ production by the substrate of *P. marginata* (92.5%) was most likely due to large amounts of fungal biomass observed in the mixed feed and substrate when insects were absent in the experiments aimed to obtain correction values for CO_2_-production by the substrate. No fungal growth was apparent during the experiments on feeding *P. marginata* larvae, suggesting that the contribution of the substrate to total respiration during the experiment was much lower. We conclude that the interaction between actively feeding *P. marginata* larvae and the substrate suppressed fungal growth through either consumption by the beetle larvae [47] of fungal biomass or through unknown chemical or combined chemical/mechanical mechanisms. Such interactions hinder the application of realistic corrections for the contribution of feed and substrate to the total CO_2_ production and thus to quantify the CO_2_ production arising from insect metabolism separately.

**Table 6:** Calculated CO_2_ production of provided feed for five insect species recalculated per kg of animal body mass.

Species	CO2 production (g)/kg BM of insect	Relative contribution
*Pachnoda marginata*	46.2	92.46%
*Tenebrio molitor*	2.2	3.58%
*Blaptica dubia*	0.4	2.31%
*Acheta domesticus*	0.9	1.34%
*Locusta migratoria*	3.3	3.04%

For all other species the relative contribution of the feed to total CO_2_ production was minor, varying between 1.3% and 3.6%. Although feed respiration did have an impact on production of CO_2_, still the production of CO_2_ is much higher for *L. migratoria* than for the other insect species. A likely explanation for this higher production of CO_2_ is the 7°C higher temperature *L. migratoria* was kept at, as a difference of 10°C is expected to double CO_2_ production. Furthermore, the comparatively high ADG of *L. migratoria* is expected to result in higher production of CO_2._

In one of the repetitions for *A. domesticus*, a lower ADG and increased mortality were observed. Excluding this repetition, the emission of CO_2_ per kg BM decreased slightly (68 vs 71 g/kg), but the emission of CO_2_ per kg mass gain changed considerably (918 vs 1468 g/kg). This difference can for a large part be explained by a decrease in ADG (from 9.0 to 7.2%). *Acheta domesticus* did not produce CH_4_, but N_2_O production doubled (from 0.1 to 0.2 mg/kg BM; 1.9 vs 5.3 mg/kg mass gain). The production of CO_2_ eq. also increased (0.04 vs 0.05 g CO_2_ eq. /kg BM and 0.57 vs 1.57 g/kg mass gain). It is well possible that the higher N_2_O production measured was caused by saprophytic bacteria utilising the dead *A. domesticus* and producing N_2_O [6]. Although we included this repetition in the results, it is not clear whether this represents the practical situation best.

Large differences in NH_3_ emission have been reported for conventional livestock. Pigs for example emit 4.8–75 mg/kg BM/day [48], [49], [50], poultry 72–436 mg/kg BM/day [41], [49], [51] and cattle 14–170 mg/kg BM/day [49], [52], [53]. Several factors influence NH_3_ emission, such as temperature, relative humidity, food type, moisture content, pH, wind speed, housing type, and substrate [54], [55].

In the current experiment, a clear NH_3_ emission pattern was found; higher amounts of NH_3_ were emitted during daytime for *A. domesticus*, *L. migratoria* and *B. dubia*, than during nighttime. Day-night rhythms for NH_3_ excretion have been documented for pigs [5] and are strongly correlated with activity levels [56]. Quantitatively the differences between day and night emission levels are small; 7–10% with a maximum difference of 25% [5]. In our study this relative difference was approximately 33%. In all cases NH_3_ emission levels were higher during the daytime than during the night-time. For *L. migratoria* this is the active period, for the nocturnal *B. dubia* and *A. domesticus* it is not, indicating that a different, unknown variable might influence NH_3_ emission patterns in these insects.

NH_3_ concentrations in the outgoing air, and consequently calculated NH_3_ emission, increased from day one to day three in *B. dubia* (1.57 to 4.29 mg/kg BM/day) and *A. domesticus* (2.46 to 8.01 mg/kg BM/day). This could indicate that NH_3_ emissions might be underestimated due to the relatively short time frame of our experiments. For *L. migratoria* NH_3_ emission did not increase between day 1 and day 3 (5.57 and 5.05 mg/kg BM/day), suggesting that NH_3_ production was stable. This might be caused by the faeces of this species that, contrary to those of *B. dubia* or *A. domesticus*, dry quickly after defecation.

We conclude that *P. marginata* and *T. molitor* probably did not emit NH_3_. Poultry deep litter systems [57] have higher NH_3_ emission rates than battery systems [55], which is explained by the presence of substrate.

The presence of substrates for *P. marginata* and *T. molitor* in this study corresponded with lower NH_3_ emissions. A possible explanation is that gas exchange in the container is inhibited by the substrate and therefore less emission of NH_3_ was measured. However, it could also be that these species produce less NH_3_.

All insect species in this study produced much lower amounts of NH_3_ (3.0 to 5.4 mg/kg BM/day for *A. domesticus, L. migratoria* and *B. dubia*) than conventional livestock (4.8–75 mg/kg BM/day for pigs and 14–170 mg/kg BM/day for cattle). Further research is needed to determine for which insect species and to what extent NH_3_ emissions increase further when a longer time frame is used.

### Conclusions

To the authors' knowledge, the study presented here is the first to report on both GHG and NH_3_ emissions of edible insect species. An evaluation of the GHG emissions of edible insect species is most relevant when based on CO_2_ eq. per kg of mass gain. In that way a comparison of the selected species with each other and with conventional livestock is based on a cost-benefit principle, in which the GHG production (environmental cost) is directly linked to food production (benefit). GHG emission of four of the five insect species studied was much lower than documented for pigs when expressed per kg of mass gain and only around 1% of the GHG emission for ruminants.

The measured NH_3_ emission levels of all insect species in this experiment were lower than reported NH_3_ emission levels for conventional livestock.

The ADG of all insect species in this study was higher than for conventional livestock, while CO_2_ production expressed as g/kg mass gain was comparable or lower, which indicates higher feed conversion efficiencies for insects.

This study therefore indicates that insects could serve as a more environmentally friendly alternative for the production of animal protein from the perspective of GHG and NH_3_ emissions. A complete lifecycle analysis for species of edible insects is lacking at this point in time [58] and should be the focus point of further studies to allow a conclusive evaluation of the sustainability of insects as a protein-rich food source. The data presented in this study are indispensable for conducting a lifecycle analysis for edible insects.
